# Evolutionary balance between LRR domain loss and young *NBS–LRR* genes production governs disease resistance in *Arachis hypogaea* cv. Tifrunner

**DOI:** 10.1186/s12864-019-6212-1

**Published:** 2019-11-13

**Authors:** Hui Song, Zhonglong Guo, Xiaohui Hu, Lang Qian, Fuhong Miao, Xiaojun Zhang, Jing Chen

**Affiliations:** 1 Grassland Agri-husbandry Research Center, College of Grassland Science, Qingdao Agricultural University, Qingdao, China; 2 State Key Laboratory of Protein and Plant Gene Research, Peking-Tsinghua Center for Life Sciences, School of Life Sciences and School of Advanced Agricultural Sciences, Peking University, Beijing, China; 3 Shandong Peanut Research Institute, Qingdao, China; 4 Dalian Academy of Agricultural Sciences, Dalian, China; 5 College of Agronomy, Qingdao Agricultural University, Qingdao, China

**Keywords:** *Arachis hypogaea* cv. Tifrunner, Genetic exchange, NBS–LRR, Selective pressure, Young gene

## Abstract

**Background:**

Cultivated peanut (*Arachis hypogaea* L.) is an important oil and protein crop, but it has low disease resistance; therefore, it is important to reveal the number, sequence features, function, and evolution of genes that confer resistance. Nucleotide-binding site–leucine-rich repeats (*NBS–LRRs*) are resistance genes that are involved in response to various pathogens.

**Results:**

We identified 713 full-length *NBS–LRRs* in *A. hypogaea* cv. Tifrunner. Genetic exchange events occurred on *NBS–LRRs* in *A. hypogaea* cv. Tifrunner, which were detected in the same subgenomes and also found in different subgenomes. Relaxed selection acted on NBS–LRR proteins and LRR domains in *A. hypogaea* cv. Tifrunner. Using quantitative trait loci (QTL), we found that *NBS–LRRs* were involved in response to late leaf spot, tomato spotted wilt virus, and bacterial wilt in *A. duranensis* (2 *NBS–LRRs*), *A. ipaensis* (39 *NBS–LRRs*), and *A. hypogaea* cv. Tifrunner (113 *NBS–LRRs*). In *A. hypogaea* cv. Tifrunner, 113 *NBS–LRRs* were classified as 75 young and 38 old *NBS–LRRs*, indicating that young *NBS–LRRs* were involved in response to disease after tetraploidization. However, compared to *A. duranensis* and *A. ipaensis*, fewer LRR domains were found in *A. hypogaea* cv. Tifrunner NBS–LRR proteins, partly explaining the lower disease resistance of the cultivated peanut.

**Conclusions:**

Although relaxed selection acted on NBS–LRR proteins and LRR domains, LRR domains were preferentially lost in *A. hypogaea* cv. Tifrunner compared to *A. duranensis* and *A. ipaensis*. The QTL results suggested that young *NBS–LRRs* were important for resistance against diseases in *A. hypogaea* cv. Tifrunner. Our results provid insight into the greater susceptibility of *A. hypogaea* cv. Tifrunner to disease compared to *A. duranensis* and *A. ipaensis*.

## Background

In plants, the innate immune system can be categorized into two layers: pattern-triggered immunity (PTI) and effector-triggered immunity (ETI) [1]. PTI is mediated by surface-localized pattern recognition receptors (PRRs) that can recognize pathogen-associated molecular patterns (PAMPs) of the pathogen. ETI is mediated by intracellular immune receptors, which evolve resistance (*R*) genes to recognize effectors of pathogens. *R* genes can be divided into at least five classes [2, 3], and the biggest category is nucleotide binding–leucine-rich repeats (*NBS–LRRs*) [4]. *NBS–LRRs* are distributed in various plant species. Many *NBS–LRRs* have been identified at the genome-wide level such as in *Arabidopsis thaliana* [5], *Arachis duranensis* [6], *Arachis ipaensis* [6], *Glycine max* [7], *Medicago truncatula* [8], *Oryza sativa* [9], and *Triticum aestivum* [10]. *NBS–LRRs* are classified into two types based on the N-terminal domain, coiled-coil (CC)–NBS–LRR (CNL) and toll/mammalian interleukin-1 receptor (TIR)–NBS–LRR (TNL) [5]. Generally, the NBS domain hydrolyzes ATP or GTP to obtain energy [2]. Overexpression of CC or TIR domains can reduce hypersensitive response in plants [11, 12]. The LRR domain undergoes more relaxed selection or positive selection because this domain interacts with pathogenic effectors [13–15], indicating that LRR domains are more diverse compared to NBS, TIR, and CC domains [13, 14, 16].

To date, a few studies have focused on the phylogenetic relationship of *NBS–LRRs* between polyploids and their donors. *T. aestivum* (AABBDD) is a hybrid of *Aegilops tauschii* (DD) and *T. dicoccoides* (AABB) which originated from a hybridization process between *T. urartu* (AA) and *A. speltoides* (BB) [17]. Many *NBS–LRRs* are extinct in *T. aestivum* compared to the *NBS–LRRs* in its donors; the evolutionary rate of *NBS–LRRs* of *T. aestivum* is also slower than that of its donors [10], causing disease resistance in *T. aestivum* to be lower than its donors. Similarly, *Gossypium hrisutum* (AADD) is a hybrid between *G. raimondii* (DD) and *G. arboretum* (AA) [18]. New *NBS–LRRs* are produced in *G. hrisutum* because of polyploidy, natural and artificial selection, gene duplication, and chromosomal recombination [19]. However, gene number and gene structure of *NBS–LRRs* are similar for *Citrus sinensis* and its donor, *C. clementina* [16]. Therefore, it is important to study the evolution and function between polyploids and parental donors.

*NBS–LRRs* involved in response to pathogens have been well documented. *RFO1*, *WRR4*, and *RPW8* genes are *NBS–LRRs* that have been isolated from *A. thaliana* [20–22]. Functional analyses have shown that *RFO1* genes provide resistance to a broad spectrum of *Fusarium* races [20], and *RPW8* controls resistance to a broad spectrum of powdery mildew pathogens [21]. Overexpression of *WRR4* in *Brassica* species can confer broad-spectrum white rust resistance [22]. In addition, a total of 15 *NBS–LRRs* from five rice cultivars have been introduced into a transgenic rice cultivar, increasing its broad-spectrum resistance to *Magnaporthe oryzae* [15]. In legumes, *RCT1* from *M. truncatula*, which is classified as a *TNL* gene, confers broad-spectrum anthracnose resistance in transgenic susceptible alfalfa plants [23]. In *Arachis*, *NBS–LRRs* are involved in response to *Aspergillus flavus* and *Meloidogyne arenaria* infection [6, 24, 25].

Cultivated peanut (*Arachis hypogaea* L., AABB) is an allotetraploid hybrid between two wild peanuts, *A. duranensis* (AA) and *A. ipaensis* (BB) [26–28]. The complete genome sequences of *A. hypogaea* cv. Tifrunner and related diploids, *A. duranensis* and *A. ipaensis*, have been published [26, 29–32]. In addition, *NBS–LRRs* of *A. duranensis* and *A. ipaensis* have been identified and subjected to phylogenetic analyses [6]. These studies provided a powerful basis for the understanding of evolution and function of *NBS–LRRs* in *A. hypogaea* cv. Tifrunner. In this study, we identified 713 full-length *NBS–LRRs* in *A. hypogaea* cv. Tifrunner. We analyzed the sequence structure, evolution and function of *NBS–LRRs* in *A. hypogaea* cv. Tifrunner. We proposed that the low disease resistance of *A. hypogaea* cv. Tifrunner may be partially caused by the loss of LRR domains.

## Results and discussion

### *NBS–LRR* gene family in *A. hypogaea* cv. Tifrunner

We identified 1105 NBS-containing sequences using HMMER in *A. hypogaea* cv. Tifrunner. Among the NBS-containing sequences, 713 NBS-containing genes contained complete NBS domains and had full-length coding sequences (Additional file 1: Table S1). Previously, results were more difficult to interpret when the evolution of NBS–LRR proteins was analyzed using the incomplete NBS domain of *Lotus japonicus* [33]. Therefore, in our study, only 713 regular *NBS–LRRs* encoding intact NBS domains were used for further analyses. There are a total of 278 and 303 full-length *NBS–LRRs* in *A. duranensis* and *A. ipaensis*, respectively [6].

Among the 713 NBS–LRR proteins, 229 sequences contained TIR domains, and 118 sequences included CC domains (Additional file 1: Table S1). Interestingly, we found that 26 sequences contained both TIR and CC domains in *A. hypogaea* cv. Tifrunner (Additional file 1: Table S1). However, none of the sequences contained both TIR and CC domains in *A. duranensis* and *A. ipaensis* [6]. Previous studies have demonstrated that TNL and CNL have different origins [34–36]. We speculated that genetic exchange or gene rearrangement likely resulted in the fusion of the TIR and CC domains after tetraploidization. Bertioli et al. [30] found many crossovers between A and B subgenomes, and chromosome inversions were detected in *A. hypogaea* cv. Tifrunner. The chromosome translacations could change gene direction. In addition, we found three sequences that simultaneously contained an NBS domain and WRKY domain in *A. hypogaea* cv. Tifrunner. In other legumes, NBS–WRKY fusion proteins have only been identified in *G. max*, *A. duranensis*, and *A. ipaensis* [37]. The bacterial effectors AvrRps4 or PopP2 can trigger WRKY transcription factors that are involved in active *NBS–LRR* gene responses to pathogens [38]. We speculated that NBS–WRKY fusion proteins can play a crucial role in response to biotic stress in *A. hypogaea* cv. Tifrunner.

LRR domains play important roles in protein–ligand and protein–protein interactions; these LRR domains are involved in plant immune responses [39, 40]. In this study, we found that 348 NBS–LRR proteins contained four types of LRR domains in *A. hypogaea* cv. Tifrunner, namely, LRR1, LRR3, LRR4, and LRR8 (Additional file 1: Table S1). Among these sequences, the greatest number of LRR domains were classified as LRR8-type (308), followed by LRR3 (133), LRR4 (88), and LRR1 (7). *A. duranensis* and *A. ipaensis* had five types of LRR domains: LRR1, LRR3, LRR4, LRR5, and LRR8 [6]. Moreover, the greatest number of LRR domains in *A. duranensis* were classified as LRR8-type, followed by LRR4, LRR3, and LRR5 [6]. In *A. ipaensis*, the greatest number of LRR domains were classified as LRR8-type, followed by LRR4, LRR3, LRR5, and LRR1 [6]. The LRR5 domain only appeared in CNL proteins in *A. duranensis* and *A. ipaensis* [6]. We proposed that *A. hypogaea* cv. Tifrunner lost the LRR5 domain possibly due to genetic exchange or gene loss after tetraploidization or whole genome duplication (WGD).

### Genetic exchange of *NBS–LRRs* in *A. hypogaea* cv. Tifrunner

**Fig. 1:**
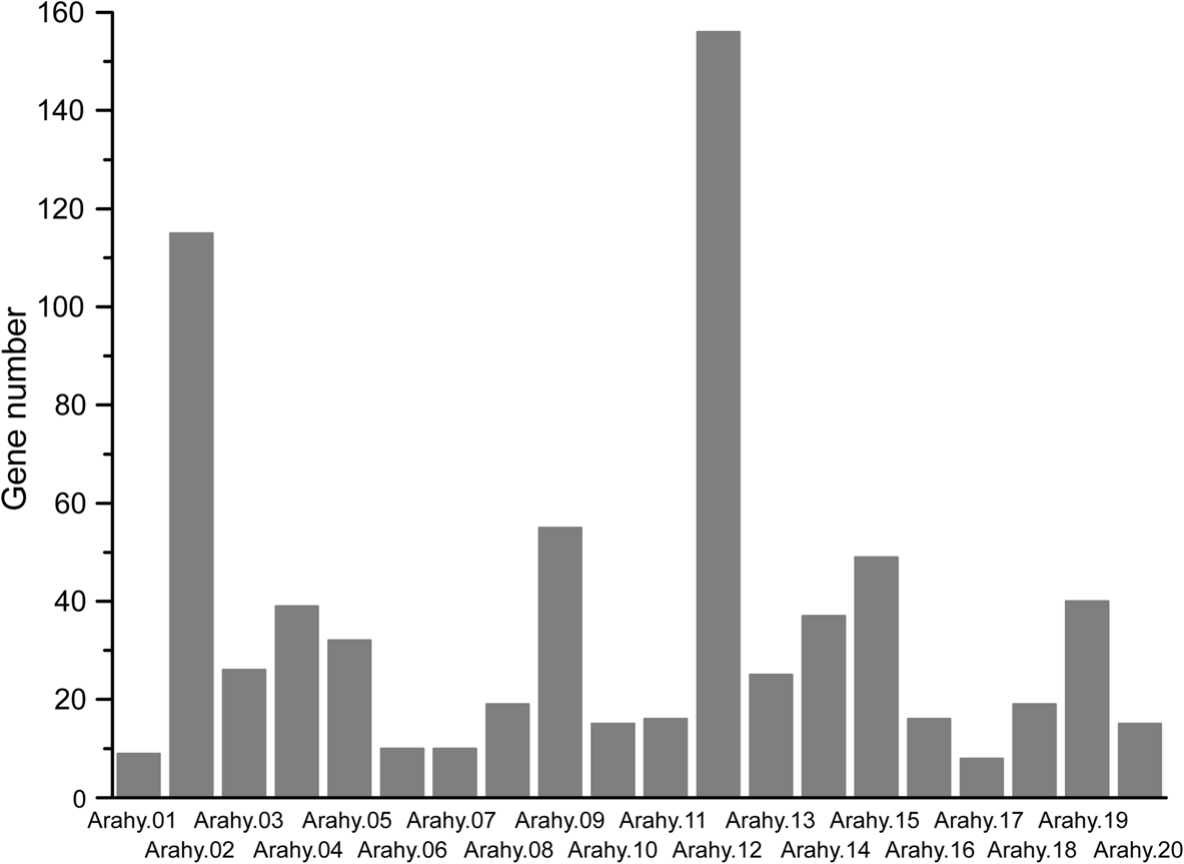
The number of *NBS–LRRs* distributed on each chromosome in *Arachis hypogaea* cv. Tifrunner

**Fig. 2:**
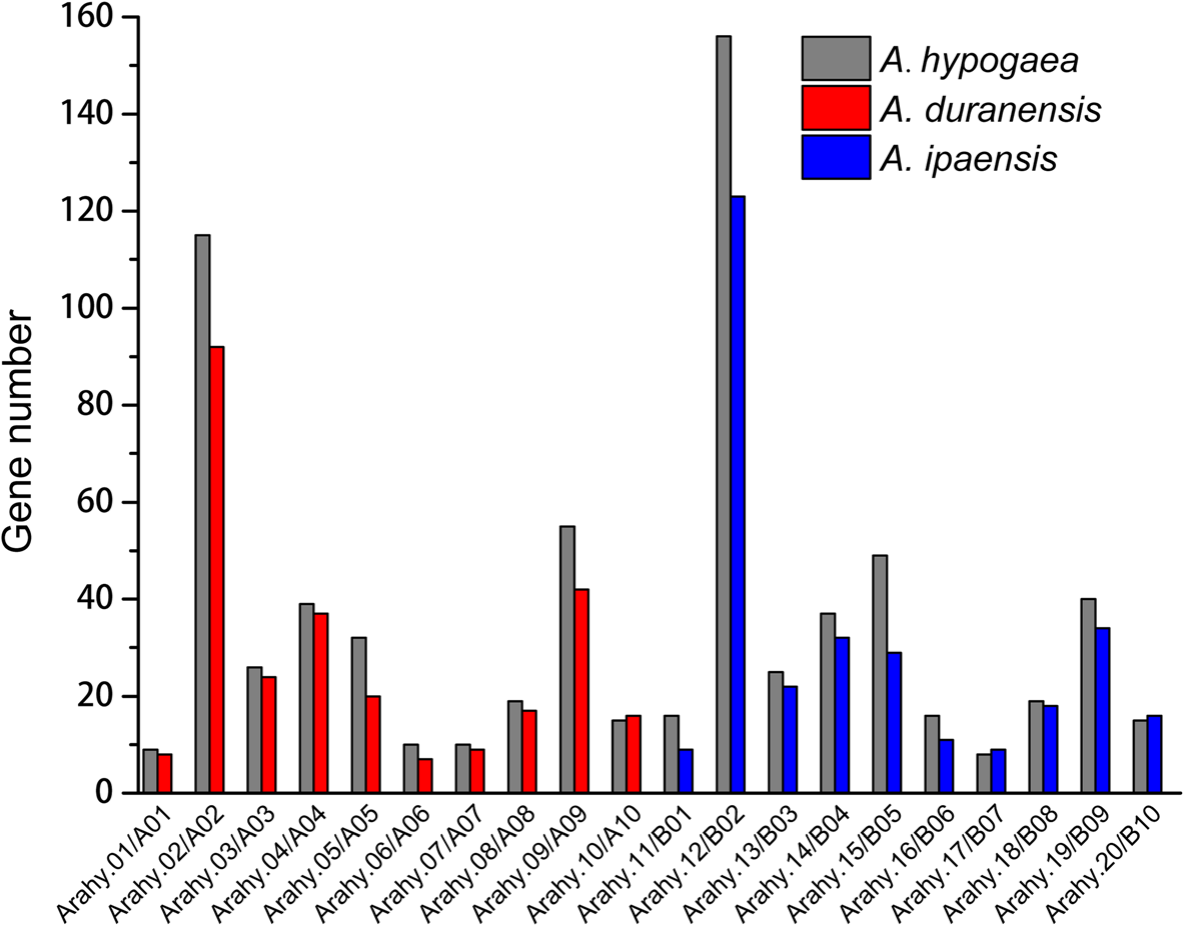
Comparison of the location of representative *NBS–LRRs* on each chromosome among *Arachis duranensis*, *A. ipaensis*, and *A. hypogaea* cv. Tifrunner

*A. hypogaea* cv. Tifrunner has 20 chromosomes, Arahy.01–Arahy.20 [30]. The chromosomal location results showed that the greatest number of *NBS–LRRs* was located on Arahy.12, while the lowest number of *NBS–LRRs* were located on Arahy.17 (Fig. 1). The chromosomal location of *NBS–LRRs* was reported in *A. duranensis* (chromosome: A01–A10) and *A. ipaensis* (chromosome: B01–B10) by Song et al. [6]. A02 and B02 contained the highest number of *NBS–LRRs* in *A. duranensis* and *A. ipaensis*, respectively, and A06 and B07 had the lowest *NBS–LRR* number in *A. duranensis* and *A. ipaensis*, respectively [6]. In this study, the A subgenome was represented as Arahy.01–Arahy.10, and B subgenome was represented as Arahy.11–Arahy.20 in *A. hypogaea* cv. Tifrunner based on the number of *NBS–LRRs* on each chromosome (Fig. 2). This result was consistent with a previous description of chromosome assembly in *A. hypogaea* cv. Tifrunner by Bertioli et al. [30].

A polyploidization event (or WGD) can cause gene duplication and loss [41, 42]. *A. hypogaea* had at least three WGDs [32]; therefore, the number of *NBS–LRRs* on each chromosome of *A. hypogaea* cv. Tifrunner changed and was different from the number of *NBS–LRRs* on each chromosome of *A. duranensis* and *A. ipaensis*. We found that although some *NBS–LRRs* were lost, the total number of *NBS–LRRs* was higher in *A. hypogaea* cv. Tifrunner. For example, the number of *NBS–LRRs* on Arahy.10, 17, and 20 decreased, and the number of *NBS–LRRs* on other chromosomes increased compared with *A. duranensis* and *A. ipaensis* (Fig. 2).

**Fig. 3:**
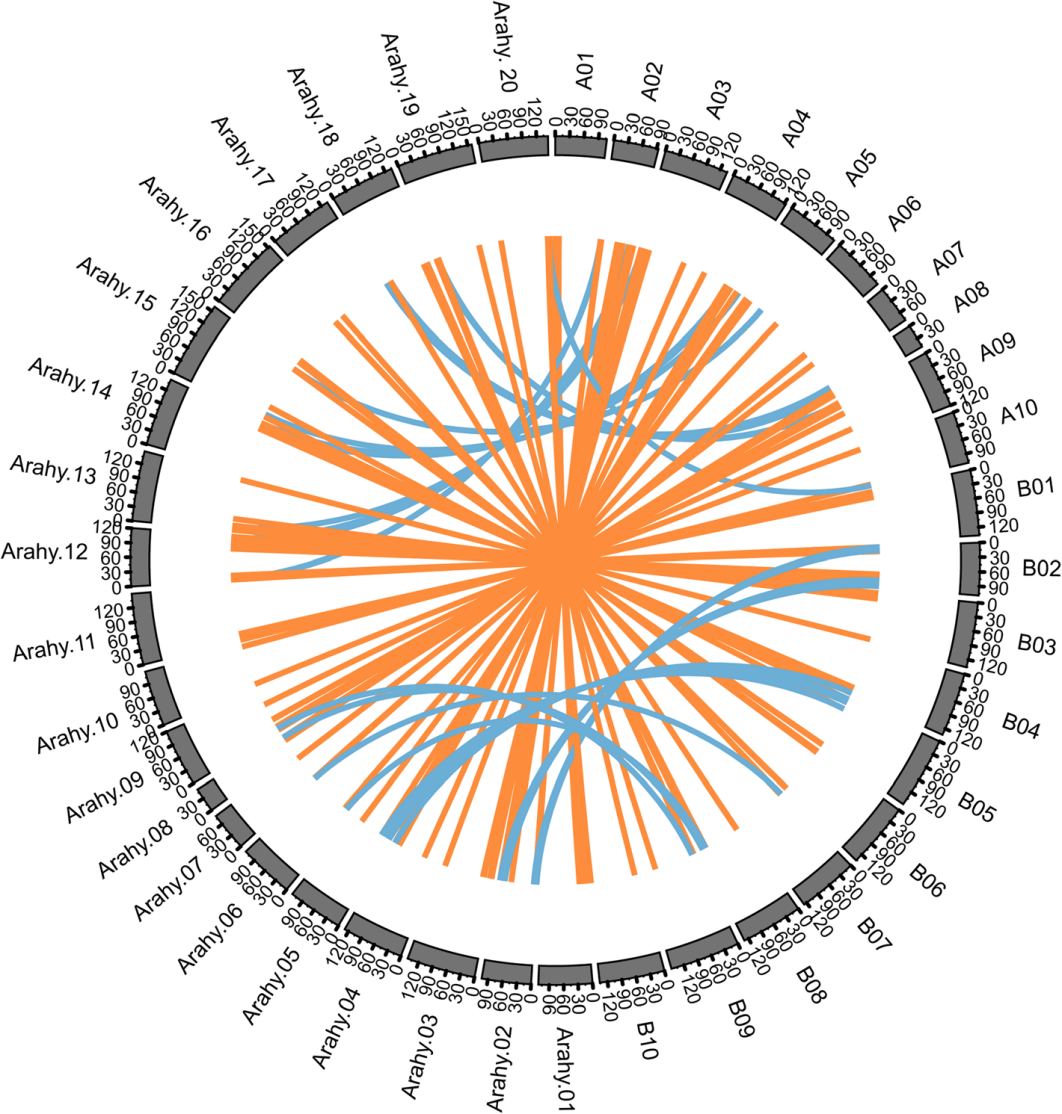
One-to-one orthologous *NBS–LRR* gene pairs among *Arachis duranensis*, *A. ipaensis*, and *A. hypogaea* cv. Tifrunner. The orange line indicates orthologous *NBS–LRR* gene pairs in a similar chromosomal location between wild and cultivated peanuts. The blue line indicates orthologous *NBS–LRR* gene pairs in a different chromosomal location between wild and cultivated peanuts

To further reveal the relationship of *NBS–LRR*s between wild and cultivated peanuts, we constructed one-to-one orthologs. A total of 99 one-to-one orthologous gene pairs were identified between *A. hypogaea* cv. Tifrunner and *A. duranensis*, and 142 one-to-one orthologous gene pairs were identified between *A. hypogaea* cv. Tifrunner and *A. ipaensis* (Fig. 3). Most one-to-one orthologs corresponded to a similar location on the chromosome between wild and cultivated peanut species. However, some *NBS–LRRs* from *A. duranensis* (A genome) corresponded to *NBS–LRRs* in the B subgenome of *A. hypogaea* cv. Tifrunner and vice versa (Fig. 3). These results indicated that there was genetic exchange in the *A. hypogaea* cv. Tifrunner genome, which is consistent with previous findings by Leal-Bertioli et al. [43], who demonstrated that *A. ipaensis* B genome segments were replaced by the *A. hypogaea* cv. Tifrunner A subgenome segments, and *A. duranensis* A genome segments were replaced by *A. hypogaea* cv. Tifrunner B subgenome segments. The genome structure was not the expected AABB, but was AAAA or BBBB in *A. hypogaea* cv. Tifrunner [30]. Specifically, approximately 14.8 Mb of the A subgenome sequences were transferred into the B subgenome, and 3.1 Mb of the B subgenome sequences migrated into the A subgenome based on genetic exchange or homoeologous exchange [30].

### Relaxed selection acting on paralogous *NBS–LRR* gene pairs in *A. hypogaea* cv. Tifrunner

**Fig. 4:**
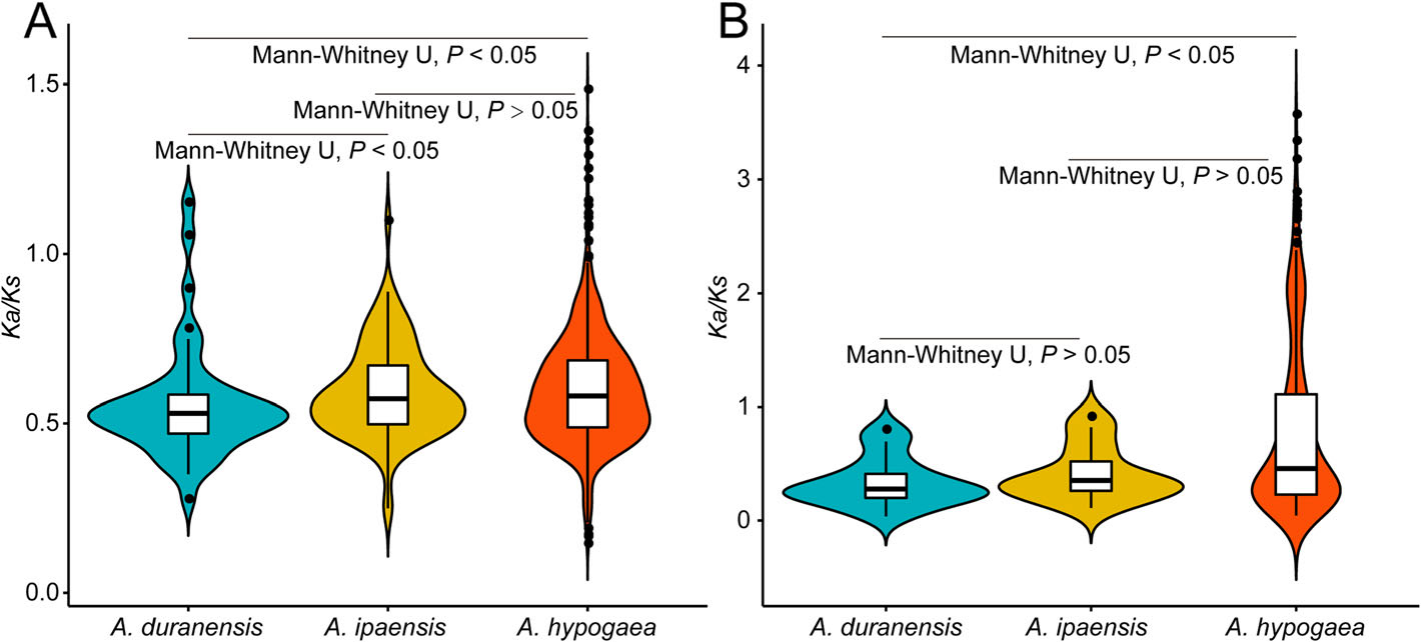
Comparison of selective pressure (*K*_a_/*K*_s_) of paralogous NBS–LRR proteins among *Arachis duranensis*, *A. ipaensis*, and *A. hypogaea* cv. Tifrunner. A. *K*_a_/*K*_s_ of paralogous NBS–LRR proteins; B. *K*_a_/*K*_s_ of paralogous LRR domains. *K*_a_/*K*_s_: nonsynonymous to synonymous per site substitution rates. *P* < 0.05 indicates a statistically significant difference

A total of 43, 87, and 756 paralogous gene pairs were found in *A. duranensis*, *A. ipaensis*, and *A. hypogaea* cv. Tifrunner, respectively (Additional file 2: Table S2 and Additional file 3: Table S3). *A. hypogaea* cv. Tifrunner had a greater number of paralogous gene pairs than *A. duranensis* and *A. ipaensis*. This could be explained by tetraploidization or WGD. Specifically, a polyploidization event may have retained many duplicated genes [41, 42]. The average *K*_*a*_*/K*_*s*_ of paralogous *NBS–LRRs* in *A. hypogaea* cv. Tifrunner (0.60) was greater than the *K*_*a*_*/K*_*s*_ of *A. ipaensis* (0.59) and *A. duranensis* (0.55, Fig. 4a). Nevertheless, the average *K*_*a*_*/K*_*s*_ value of paralogous *NBS–LRRs* was greater than 0.5 in *A. duranensis*, *A. ipaensis*, and *A. hypogaea* cv. Tifrunner, indicating that the paralogous *NBS-LRRs* were under relaxed selection.

Compared to other domains of NBS–LRR proteins, the LRR domain underwent more relaxed selection or positive selection because this domain was implicated in pathogenic effector sensing [13–15]. Our results showed that the average *K*_*a*_*/K*_*s*_ value of the LRR domain in *A. hypogaea* cv. Tifrunner (0.80) was greater the average *K*_*a*_*/K*_*s*_ value of *A. duranensis* (0.33) and *A. ipaensis* (0.41, Fig. 4b), suggesting that LRR domains were under relaxed selection in *A. hypogaea* cv. Tifrunner, but under purifying selection in *A. duranensis* and *A. ipaensis*.

### Young *NBS–LRR* paralogs in *A. hypogaea* cv. Tifrunner

**Fig. 5:**
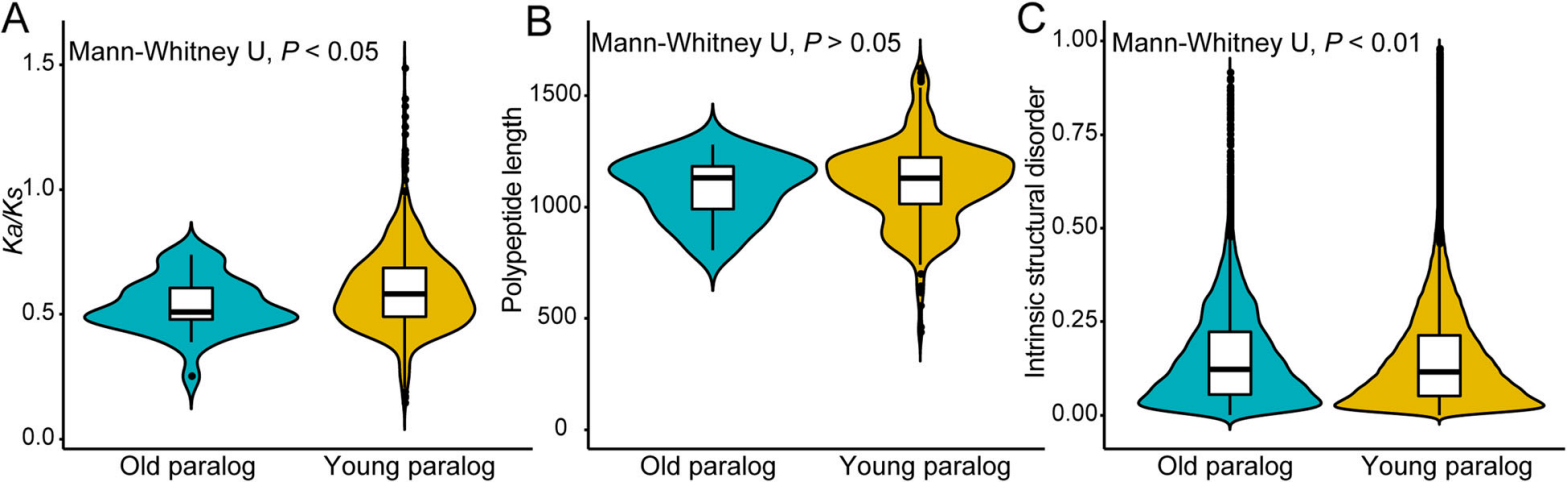
Comparison of sequence features and substitution rates between old and young paralogous NBS–LRR proteins in *Arachis hypogaea* cv. Tifrunner. A. Selective pressure (*K*_a_/*K*_s_) between old and young paralogous NBS–LRR proteins in *A. hypogaea* cv. Tifrunner; B. Polypeptide length between old and young paralogous NBS–LRR proteins in *A. hypogaea* cv. Tifrunner; C. The intrinsic structural disorder (ISD) of old and young paralogous NBS–LRR proteins in *A. hypogaea* cv. Tifrunner. *K*_a_/*K*_s_: nonsynonymous to synonymous per site substitution rates. *P* < 0.05 and < 0.01 indicate significant differences

In this study, the paralogs produced by gene duplication events that occurred before tetraploidization were considered old paralogs. Young paralogs were generated by gene duplication events after tetraploidization. We detected 29 old and 727 young paralogous *NBS–LRR* gene pairs in *A. hypogaea* cv. Tifrunner (Additional file 3: Table S3), indicating that many young *NBS–LRR* paralogs were generated as a result of gene duplication events after tetraploidization. In addition, some old paralogous *NBS–LRR* gene pairs were lost after tetraploidization, where A subgenome lost 35 paralogous *NBS–LRR* gene pairs, and B subgenome lost 66 paralogous *NBS–LRR* gene pairs compared with *A. duranensis* and *A. ipaensis*. Previous studies have reported that the properties of old and young genes have different features [44–50]. For example, young genes have faster evolutionary rates, relaxed selection, lower gene expression levels, shorter gene length, and higher intrinsic structural disorder (ISD) than old genes [46, 47, 49–53]. We found that the average *K*_*a*_*/K*_*s*_ values of young paralogous *NBS–LRRs* (0.60) were higher than old *NBS–LRRs* (0.54, Fig. 5a), indicating that young paralogous *NBS–LRRs* were under relaxed selection. The average polypeptide length of young paralogous *NBS–LRRs* (1110 amino acids) was longer than old paralogous *NBS–LRRs* (1080 amino acids; Fig. 5b). The average ISD value of young paralogous *NBS–LRRs* (0.14) was lower than the old paralogous *NBS–LRRs* (0.15, Fig. 5c), indicating that the protein structure of young paralogous *NBS–LRRs* was stable compared to old paralogous *NBS–LRRs*. In contrast to these findings, previous studies have found that young genes often have shorter gene length and higher ISD compared to old genes [46, 49]. Young gene has essential function at least underwent 100 MYA [52]. However, the *A. hypogaea* origination is relatively late [26, 31]. Therefore, we speculated that young *NBS–LRRs* played the essential functions need more time, it was just rapidly fixed in *A. hypogaea* cv. Tifrunner.

### NBS–LRR proteins lost LRR domains in *A. hypogaea* cv. Tifrunner

**Fig. 6:**
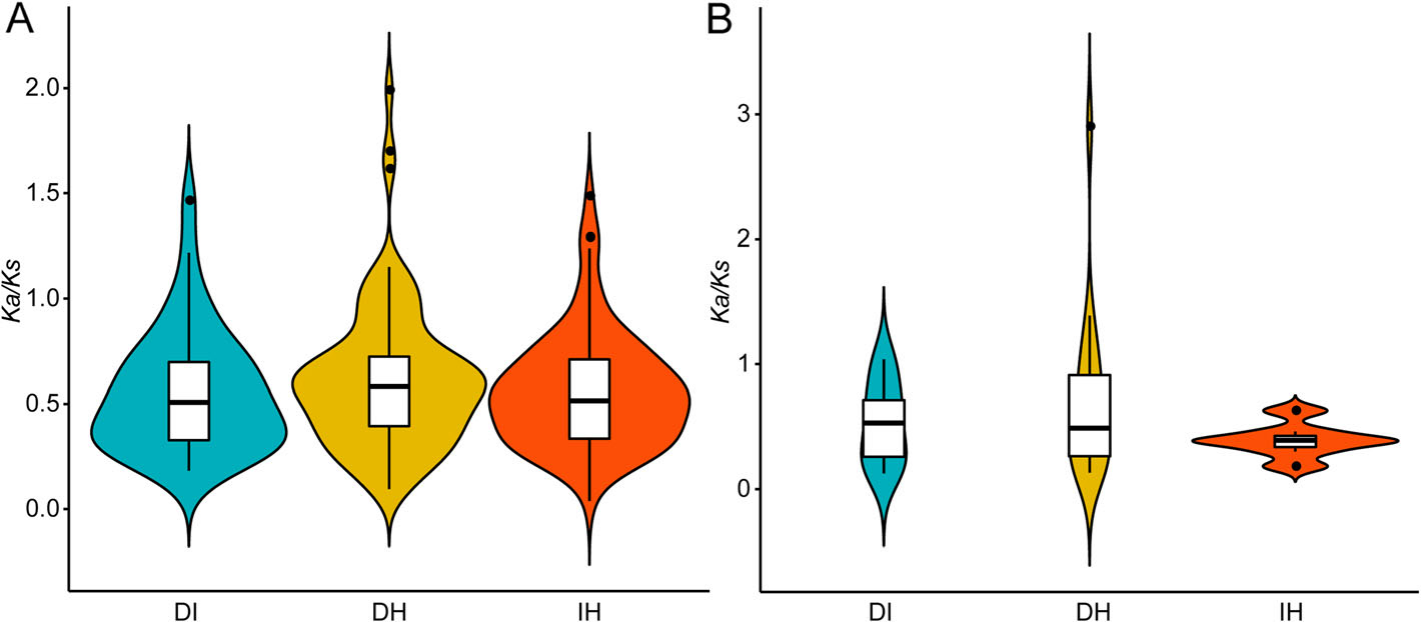
Comparison of selective pressure (*K*_a_/*K*_s_) between orthologous NBS–LRR proteins among *Arachis duranensis*, *A. ipaensis*, and *A. hypogaea* cv. Tifrunner. A. *K*_a_/*K*_s_ of orthologous NBS–LRR proteins; B. *K*_a_/*K*_s_ of orthologous LRR domains. DI. *A. duranensis* VS *A. ipaensis*; DH. *A. duranensis* VS *A. hypogaea* cv. Tifrunner; IH. *A. ipaensis* VS *A. hypogaea* cv. Tifrunner. *K*_a_/*K*_s_: nonsynonymous to synonymous per site substitution rates

**Fig. 7:**
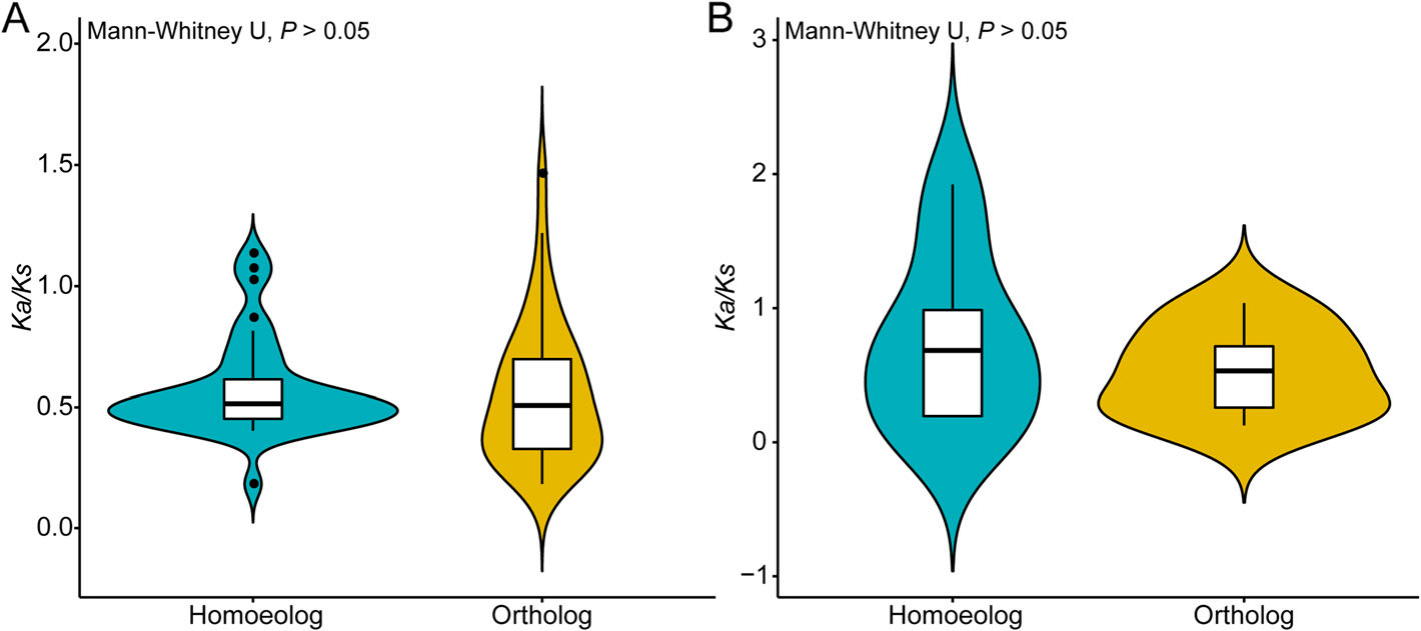
Comparison of selective pressure (*K*_a_/*K*_s_) between homoeologous NBS–LRR proteins and orthologous NBS–LRR proteins among *Arachis duranensis*, *A. ipaensis*, and *A. hypogaea* cv. Tifrunner. A. *K*_a_/*K*_s_ of NBS–LRR proteins; B. *K*_a_/*K*_s_ of LRR domains. *K*_a_/*K*_s_: nonsynonymous to synonymous per site substitution rates

NBS–LRR orthologs in *A. duranensis*, *A. ipaensis*, and *A. hypogaea* cv. Tifrunner were under relaxed selection (Fig. 6a), indicating that the biological functions of NBS–LRRs diversified after the divergence of these three *Arachis* species. Relaxed selection acted on LRR domains of NBS–LRR orthologs between *A. duranensis* and *A. ipaensis* (0.53) and between *A. duranensis* and *A. hypogaea* cv. Tifrunner (0.71) and purifying selection acted on LRR domains from NBS–LRR orthologs between *A. ipaensis* and *A. hypogaea* cv. Tifrunner (0.39; Fig. 6b). These results indicated that the LRR domains between *A. ipaensis* and *A. hypogaea* cv. Tifrunner were conserved, and LRR domains between *A. duranensis* and *A. hypogaea* cv. Tifrunner were divergent. Moreover, we found that the average *K*_*a*_*/K*_*s*_ value of homoeologous NBS–LRR proteins (0.57) and LRR domains (0.75) in *A. hypogaea* cv. Tifrunner was greater than the average *K*_*a*_*/K*_*s*_ value of orthologs between *A. duranensis* and *A. ipaensis* (NBS–LRR: 0.55; LRR domain: 0.53; Fig.7). Taken together, the LRR domains were under more relaxed selection after tetraploidization.

**Fig. 8:**
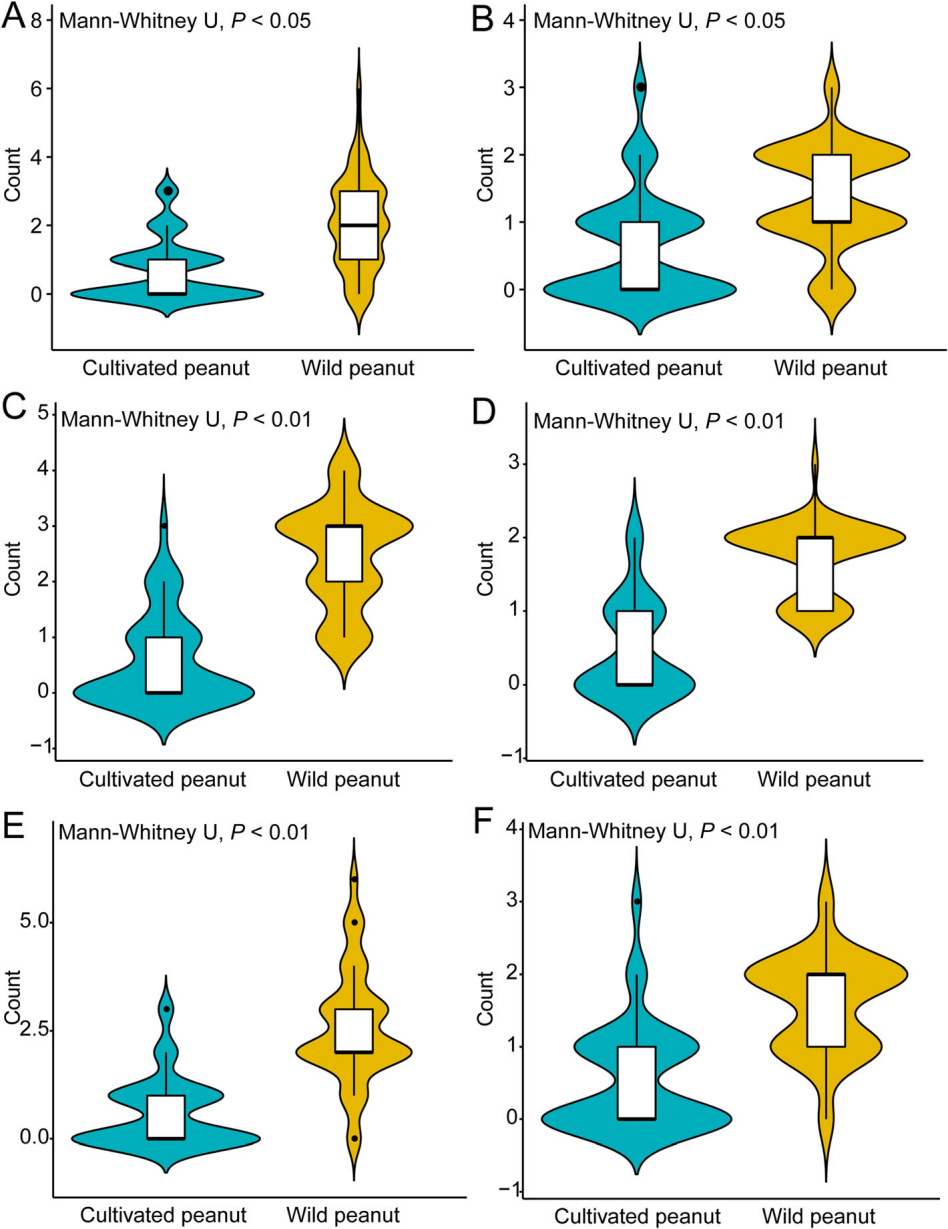
Comparison of number and type of LRR domains between wild and cultivated peanuts. A. Number of LRR domains between wild and cultivated peanuts; B. Type of LRR domains between wild and cultivated peanuts. C. Number of LRR domains between homoeologous NBS–LRRs and its orthologs; D. Type of LRR domains between homoeologous NBS–LRRs and its orthologs. E. Number of LRR domains from NBS–LRRs that respond to late leaf spot, tomato spotted wilt virus, and bacterial wilt between wild and cultivated peanuts. F. Type of LRR domains from NBS–LRRs that respond to late leaf spot, tomato spotted wilt virus, and bacterial wilt between wild and cultivated peanuts. *P* < 0.05 and < 0.01 indicate statistical significant differences

The number of LRR domains in *A. duranensis* and *A. ipaensis* were greater than that in *A. hypogaea* cv. Tifrunner (average number: 2.35 vs 0.72; Fig. 8a). There were fewer types of LRR domains in *A. hypogaea* cv. Tifrunner NBS–LRRs compared to *A. duranensis* and *A. ipaensis* (average number of type: 1.45 vs 0.64; Fig. 8b). Similarly, the number of LRR domains in orthologs of *A. duranensis* and *A. ipaensis* was greater than the homoeologs of *A. hypogaea* cv. Tifrunner (average number: 2.48 vs 0.56, average number of type: 1.73 vs 0.48; Fig. 8c and d).

Although relaxed selection had a greater effect on the NBS–LRRs of *A. hypogaea* cv. Tifrunner compared to *A. duranensis* and *A. ipaensis*, *A. hypogaea* cv. Tifrunner lost a greater number of LRR domains. These results indicated that the resistance of *A. hypogaea* cv. Tifrunner to biotic effectors was weaker than that of *A. duranensis* and *A. ipaensis,* likely because *A. hypogaea* cv. Tifrunner lost LRR domains. Similarly, Peele et al. [54] found that *A. thaliana* was sensitive to biotic stress due to the loss of LRR domains compared to *Arabidopsis lyrata*, *Capsella rubella*, *Brassica rapa*, and *Eutrema salsugineum*.

It is unclear whether *A. duranensis* donated the A subgenome to *A. hypogaea* [26]. A recent study showed that the genome of *A. duranensis* from Rio Seco, Argentina, was the most similar to the A subgenome of *A. hypogaea* using chloroplast and ribosomal DNA haplotypes from 50 accessions [30]. In this study, we used *A. duranensis* (no. V14167) from Argentina [26]. Although there may be differences in the species used in this study, our data suggests that these potential population-level differences did not influence our results. The A subgenome from *A. hypogaea* had an average DNA similarity of 99.76% to the *A. duranensis* Rio Seco accessions and 99.61% similarity to *A. duranensis* V14167 using whole-genome sequencing [30].

### *NBS–LRRs* involved in biotic resistance based on QTLs in *A. hypogaea* cv. Tifrunner

The QTLs of resistance to late leaf spot, tomato spotted wilt virus, and bacterial wilt were identified in cultivated peanut using *A. duranensis* and *A. ipaensis* as reference genomes [55, 56]. Three QTLs with 27 *NBS–LRRs*, four QTLs with six *NBS–LRRs*, and one QTL with eight *NBS–LRRs* were involved in response to late leaf spot, tomato spotted wilt virus, and bacterial wilt, respectively (Table 1 and Additional file 4: Table S4). All of these QTLs were mapped onto the genome of *A. hypogaea* cv. Tifrunner. One QTL (qTSW_T10_B03_1) contained two *NBS–LRRs* in *A. ipaensis*, but its collinear region was absent in *NBS–LRRs* in *A. hypogaea* cv. Tifrunner (Table 1), indicating that some *NBS–LRRs* were lost in *A. hypogaea* cv. Tifrunner.

**Table 1:** The number of *NBS–LRRs* in QTLs that respond to late leaf spot, tomato spotted wilt virus, and bacterial wilt in *Arachis duranensis*, *A. ipaensis*, and *A. hypogaea* cv. Tifrunner

QTLs in wild peanut^a^	Genomic region (bp)^b^	NO. *NBS–LRR* in wild peanut	QTLs in cultivated peanut^c^	Genomic region (bp)^d^	NO. *NBS-LRR* in cultivated peanut
qLLS_T12_A05_5	15,720,064–42,599,528	2	qLLS_T12_Arahy05_5	40,799,649–18,809,983	3
qLLS_T11_B02_1	105,499,048–106,618,489	21	qLLS_T11_Arahy02_1	117,079,303–118,213,823	25
qLLS_T12_B10	10,864,883–11,224,499	4	qLLS_T12_Arahy20	11,390,610–11,757,408	3
qTSW_T10_B02	99,031,265–101,253,445	1	qTSW_T10_Arahy12	110,327,651–112,677,850	4
qTSW_T10_B03_1	128,864,060–128,903,550	2	qTSW_T10_Arahy13_1	139,479,956–139,524,916	0
qTSW_T10_B09_1	9,631,598–14,497,666	1	qTSW_T10_Arahy19_1	9,479,684–14,682,777	1
qTSW_T10_B09_2	6,739,506–5,189,475	2	qTSW_T10_Arahy19_2	6,650,549–4,973,413	6
qBWR_Com_B02	3,250,000–6,600,000	8	qBWR_Com_Arahy12	461,172–7,066,164	71

Note: QTLs: quantitative trait locus

^a^ The QTLs are named from references 55 and 56. A and B indicated the chromosome in *A. duranensis* and *A. ipaensis*, respectively

^b^ The genomic region of QTLs located on *A. duranensis* and *A. ipaensis*

^c^ The QTLs named based on the collinear region between wild and cultivated peanuts. ‘Arahy’ indicates the chromosome in *A. hypogaea* cv. Tifrunner

^d^ The genomic region of QTLs located on *A. hypogaea* cv. Tifrunner

In the collinear region, *A. duranensis* and *A. ipaensis* had greater number of LRR domains than *A. hypogaea* cv. Tifrunner (average number: 2.56 vs 0.60, average number of type: 1.58 vs 0.56; Fig. 8e and f). These results indicated that the loss of LRR domains may have decreased ability of NBS-LRR to recognize effectors of bacterial wilt, late leaf spot, and tomato spotted wilt virus in *A. hypogaea* cv. Tifrunner. Many studies have demonstrated that *A. duranensis* and *A. ipaensis* have greater resistant to biotic stressors than cultivated peanut [57–60]. Thus, we proposed that we may have overestimated the disease resistance of cultivated peanut using *A. duranensis* and *A. ipaensis* as reference genomes.

In this study, we identified 31, 11, and 71 *NBS–LRRs* that responded to late leaf spot, tomato spotted wilt virus, and bacterial wilt in *A. hypogaea* cv. Tifrunner, respectively. Among these *NBS–LRRs*, we found 75 young *NBS–LRRs* and 38 old *NBS–LRRs* based on gene duplication events after tetraploidization. There were more young *NBS–LRRs* compared to old *NBS–LRRs* in *A. hypogaea* cv. Tifrunner, indicating that young *NBS–LRRs* were involved in the plant’s response against pathogens. Similarly, Song et al. [61] found that compared to old duplicated genes, young duplicated genes were more likely to be involved in response to biotic stressors in *A. duranensis*. Although no studies have demonstrated that young genes confer resistance to biotic stress in *A. hypogaea* cv. Tifrunner, our results indicated that young *NBS–LRRs* may be involved in response to late leaf spot, tomato spotted wilt virus, and bacterial wilt compared to old *NBS–LRRs* in *A. hypogaea* cv. Tifrunner.

## Conclusions

We identified *NBS–LRRs* in *A. hypogaea* cv. Tifrunner. Genetic exchange events occurred in *NBS-LRRs* in *A. hypogaea* cv. Tifrunner compared to *A. duranensis* and *A. ipaensis*. Although the LRR domains were under relaxed selection, more LRR domains were lost in *A. hypogaea* cv. Tifrunner compared to *A. duranensis* and *A. ipaensis*. Based on the QTL data, we found that *NBS–LRRs* were involved in response to late leaf spot, tomato spotted wilt virus, and bacterial wilt in *A. duranensis*, *A. ipaensis*, and *A. hypogaea* cv. Tifrunner. Interestingly, the results suggested that young *NBS–LRRs* were more likely to be involved in disease resistance compared to old *NBS-LRRs* in *A. hypogaea* cv. Tifrunner.

## Methods

### Identification of the *NBS–LRR* gene family in *A. hypogaea* cv. Tifrunner

The complete genome sequence of *A. hypogaea* cv. Tifrunner has been published [30] and is available on PeanutBase (https://www.peanutbase.org/data/public/Arachis_hypogaea/) [29]. The hidden Markov models (HMM) of NBS (PF00931) and TIR (PF01582) domains were downloaded from the Pfam database [62]. We identified the NBS-containing sequences using NBS domain by HMMER [63] in *A. hypogaea* cv. Tifrunner. We extracted NBS-containing sequences using an in-house Perl script based on the sequencing ID. Subsequently, we uploaded the NBS-containing sequences to the Pfam database [62] and re-examined these sequences. Among the NBS-containing sequences, we used the same method to identify the TIR-containing sequences. In *A. duranensis* and *A. ipaensis*, we found the following five types of LRR domains: LRR1, LRR3, LRR4, LRR5, and LRR8 [6]. We downloaded these five HMMs of the LRR domain from the Pfam database [62] and identified the LRR domains in NBS-containing sequences using HMMER [63] in *A. hypogaea* cv. Tifrunner. The CC domains of NBS-containing sequences were surveyed using Paircoil2 (http://groups.csail.mit.edu/cb/paircoil2/). The *P*-score cutoff was 0.03.

### Chromosomal location

The gff3 file of the *A. hypogaea* cv. Tifrunner genome has been released on PeanutBase (https://www.peanutbase.org/data/public/Arachis_hypogaea/) [29]. We used the TBtools program [64] to extract the chromosomal location of *NBS–LRRs* based on the sequencing ID. The chromosomal location of *NBS–LRRs* was reported in *A. duranensis* and *A. ipaensis* [6]. We used Circos v0.69 [65] to compare the chromosomal location of *NBS–LRRs* in *A. duranensis*, *A. ipaensis*, and *A. hypogaea* cv. Tifrunner.

### Homology in *Arachis* species

Genes that are paralogs and orthologs in *A. duranensis* and *A. ipaensis* have been reported in previous studies [66, 67]. We identified *NBS–LRR* paralogs and homoeologs in *A. hypogaea* cv. Tifrunner, and *NBS–LRR* orthologs between wild and cultivated peanut species. The following evaluation criteria were used as thresholds to determine paralogs and homoeologs in local BLAST analyses [26]: (1) alignment coverage exceeding 80% of the two sequences, (2) identity > 80%, and (3) E-value ≤10^− 10^.

The paralogous, orthologous, and homoeologous *NBS–LRR* gene pairs were extracted using an in-house Perl script. MAFFT [68] was used to align pairs of amino acid sequences. PAL2NAL [69] was used to convert amino acid sequences into their corresponding nucleotide sequences. PAML 4.0 [70] was used to calculate the nonsynonymous substitution per nonsynonymous site (*K*_*a*_), synonymous substitution per synonymous site (*K*_*s*_), and nonsynonymous to synonymous per site substitution rates (*K*_a_/*K*_s_). *K*_a_/*K*_s_ = 1, *K*_a_/*K*_s_ > 1, and *K*_a_/*K*_s_ < 1 indicated neutral, positive, and purifying selection, respectively. We estimated the *K*_s_, *K*_a_, and *K*_a_/*K*_s_ of LRR domains using the same methods.

### Polypeptide length and intrinsic structural disorder

The polypeptide length of each NBS–LRR sequence was estimated using codon W (version 1.4, http://codonw.sourceforge.net) with default parameters. The intrinsic structural disorder (ISD) was estimated using IUPred2A with default parameters [71]. The ISD value ranged from 0 to 1, where 0 indicated a stable protein structure, and 1 indicated an unstable protein structure.

### Identification of the potential function of *NBS–LRRs* using quantitative trait loci analysis

To date, many recombinant inbred peanut lines have been constructed to improve biotic resistance, including resistance to bacterial, fungal, insect, and viral stressors. A number of major quantitative trait loci (QTL) were obtained using various molecular markers and genome sequencing methods [55, 56, 72–75]. Agarwal et al. [55] identified major QTLs related to response to early leaf spot, late leaf spot, and tomato spotted wilt virus using a recombinant inbred population (Tifrunner × GT-C20). Luo et al. [56] identified two QTLs that act in response to bacterial wilt using a recombinant inbred population (Yuanza 9102 × Xuzhou 68–4). The abovementioned QTLs were obtained using genome sequencing of *A. duranensis* and *A. ipaensis* as the reference genomes [55, 56]. We obtained these QTLs, and mapped them onto the genome sequences of *A. hypogaea* cv. Tifrunner using a local BLAST program [76]. The parameters were set as follows: (1) alignment coverage exceeding 80% of QTL sequences, (2) identity > 80%, and (3) E-value ≤10^− 10^. The *NBS–LRRs* were identified using the gene location information across the collinear areas in *A. duranensis*, *A. ipaensis*, and *A. hypogaea* cv. Tifrunner.

## Supplementary information

**Additional file 1: Table S1.** Information of chromosomal location and structure in *Arachis hypogaea* cv. Tifrunner *NBS–LRRs*.

**Additional file 2: Table S2.** The paralogous *NBS–LRRs* in *Arachis duranensis*, *A. ipaenesis*. MAFFT was used to align amino acid sequence pairs. PAL2NAL was used to convert amino acid sequences into the corresponding nucleotide sequences. PAML 4.0 was used to calculate the nonsynonymous substitution per nonsynonymous site (*K*_*a*_), synonymous substitution per synonymous site (*K*_*s*_), and nonsynonymous to synonymous per site substitution rates (*K*_a_/*K*_s_).

**Additional file 3: Table S3.** The paralogous *NBS–LRRs* in *A. hypogaea* cv. Tifrunner. MAFFT was used to align amino acid sequence pairs. PAL2NAL was used to convert amino acid sequences into the corresponding nucleotide sequences. PAML 4.0 was used to calculate the nonsynonymous substitution per nonsynonymous site (*K*_*a*_), synonymous substitution per synonymous site (*K*_*s*_), and nonsynonymous to synonymous per site substitution rates (*K*_a_/*K*_s_).

**Additional file 4: Table S4.** The *NBS–LRRs* identified in each QTL in *Arachis duranensis*, *A. ipaenesis*, and *A. hypogaea* cv. Tifrunner. QTL: quantitative trait loci. ^a^ The QTLs are named from references 55 and 56. A and B indicated the chromosome in *A. duranensis* and *A. ipaensis*, respectively. ^b^ The genomic region of QTLs located on *A. duranensis* and *A. ipaensis*.

## Data Availability

The datasets used and/or analyzed during the current study are available from the corresponding author on reasonable request.

## Abbreviations

CC: Coiled-coil
HMM: Hidden Markov models
ISD: Intrinsic structural disorder
*K*_a_: Nonsynonymous substitution per nonsynonymous site
*K*_a_/*K*_s_: Nonsynonymous to synonymous substitution ratio
*K*_s_: Synonymous substitution per synonymous site
NBS–LRR: Nucleotide-binding site–leucine-rich repeat
QTL: Quantitative trait loci
TIR: Toll/mammalian interleukin-1 receptor
WGD: Whole-genome duplication
